# Initial Real-World Experience of Tricuspid Transcatheter Edge-to-Edge Repair in Asia

**DOI:** 10.1016/j.jacasi.2025.07.024

**Published:** 2025-09-24

**Authors:** Kent Chak-yu So, Krissada Meemook, Jianqiang Xu, Chun-chin Chang, Tawai Ngernsritrakul, Surakiat Leelasithorn, Ching-wei Lee, Angel Lai, Kevin Ka-ho Kam, Bryan P. Yan, Alex Pui-wai Lee, Adam S.H. Sung, Yat-yin Lam

**Affiliations:** a Division of Cardiology, Department of Medicine and Therapeutics, The Chinese University of Hong Kong, Hong Kong SAR, China; b Li Ka Shing Institutes of Health Science, Prince of Wales Hospital, The Chinese University of Hong Kong, Hong Kong SAR, China; c Division of Cardiology, Department of Medicine, Faculty of Medicine, Ramathibodi Hospital, Mahidol University, Bangkok, Thailand; d Heart and Vascular Institute, The Chinese University of Hong Kong, Hong Kong SAR, China; e Cardiovascular Research Center, College of Medicine, National Yang Ming Chiao Tung University, Taipei, Taiwan; f Central Health Medical Practice, Hong Kong SAR, China

**Keywords:** Asia, combined procedures, transcatheter edge-to-edge repair, tricuspid regurgitation

## Abstract

**Background:**

Tricuspid regurgitation (TR) causes significant morbidity. Transcatheter edge-to-edge repair (TEER) has been proven effective in relieving TR symptoms. Access to tricuspid TEER and the associated experience are limited in Asia.

**Objectives:**

This study aimed to summarize the initial experience with the tricuspid TEER system (Abbott) in Asia.

**Methods:**

Patients undergoing tricuspid TEER (Abbott) from 2017 to 2024 were enrolled from 4 centers in Asia. The primary endpoint was device success (TR ≤moderate) at 30 days. Secondary endpoints included inpatient complications, 30-day NYHA functional class, and 30-day major adverse events. Risk factors for 30-day device success were identified.

**Results:**

A total of 106 patients were included, with a mean age of 76.1 ± 10 years, and 88.7% (n = 94 of 106) had atrial fibrillation. Most TR cases treated were functional (88.7% [n = 94 of 106]), with over half classified as massive or torrential (56.6% [n = 60 of 106]). Tricuspid TEER was performed using off-label MitraClip in 22.6% (n = 24), while the remaining 77.4% (n = 82) utilized TriClip. Combined procedures (with mitral TEER and others) were common (47.2% [n = 50 of 106]). At 30 days, 74.0% (n = 77 of 104) achieved device success (TR ≤moderate), and 95.2% (n = 99 of 104) experienced at least a 1-grade TR reduction. Additionally, 96.2% (n = 100 of 104) were NYHA functional class I/II, and major adverse events were 1.9% (n = 2 of 106) at 30 days. Baseline nontorrential TR and increased clinical experience (second half of cases) were associated with 30-day device success (*P =* 0.010 and *P =* 0.044, respectively).

**Conclusions:**

The early experience with tricuspid TEER in Asia is promising, demonstrating a reasonable device success rate and a high safety profile. Clinical experience is associated with improved device success.

Tricuspid regurgitation (TR) is associated with significant morbidity and mortality, creating a substantial clinical and economic burden,^1^ particularly in the Asia Pacific (APAC) region.^2^ While surgical options for isolated TR remain limited due to high operative risks, transcatheter tricuspid valve interventions have emerged as a promising alternative. Mitral transcatheter edge-to-edge repair (TEER) is now an established therapy in APAC for treating severe mitral regurgitation (MR), with promising data supporting its efficacy and safety in the Asian population.^3^^,^^4^ Like most other regions, tricuspid TEER began with the off-label MitraClip device (Abbott) in APAC in 2017.^5^ However, subsequent adoption has been very slow due to limited device availability, slow regulatory processes, and a lack of reimbursement policies across APAC countries.^2^ Only in recent years have clinicians in the APAC region increasingly utilized new transcatheter tricuspid technologies.^2^^,^^6^ Yet, data on tricuspid TEER in the APAC region are limited. This study aims to address the current gap in real-world data by reporting the initial experience and 30-day outcomes of tricuspid TEER in the APAC region.

## Methods

All consecutive tricuspid TEER procedures performed for severe symptomatic TR at 4 major TEER centers in Asia from 2017 to 2024 were included in the study. The study was conducted in accordance with the Declaration of Helsinki, and the protocol was approved by the local ethics committees.

Patients’ demographics, perioperative laboratory results, and medication records were recorded. Preoperative, intraoperative, and postoperative echocardiograms were reviewed at individual sites to determine left heart function, severity and mechanism of MR, and severity, mechanism, and location of TR, as well as the location of the clip applied. Procedural records were reviewed to determine the type of procedures performed, the number and type of clips applied, and the procedural time.

### Definitions

Severity of TR is classified into 5 grades (mild: 1+; moderate: 2+; severe: 3+; massive: 4+; torrential: 5+).^7^ Mechanisms of TR were classified into functional TR, organic TR, and cardiac implantable electronic device (CIED) associated. Additionally, the locations of TR were categorized into predominantly anteroseptal (AS), posteroseptal (PS), and diffuse (ie, throughout).^8^ Clipping strategies were classified into single-clip technique (at AS or PS), zipping technique (multiple clips placed at AS or PS alone), or clover technique (multiple clips placed at both AS and PS).

### Endpoints

Device success was defined as TR ≤moderate.^9^ The primary endpoint was device success at 30 days.^9^ Secondary endpoints included inpatient complications, TR reduction by at least 1 grade,^10^ TR reduction by at least 2 grades, 30-day NYHA functional class, and 30-day major adverse events according to the Tricuspid Valve Academic Research Consortium^11^ and CIED lead functions after tricuspid TEER. Associated risk factors for 30-day device success were identified. Specifically, patients were divided into first half vs second half at each site to assess the impact of clinical experience to 30-day device success. A comparison of patient profiles and procedural outcomes with published TriClip registries^10^^,^^12^ from Europe and the United States was performed.

### Statistical analysis

Parametric data were presented as mean ± SD, and nonparametric data were presented as median (IQR). Differences were determined by 2-sample Student’s *t* tests or Mann-Whitney *U* tests, respectively. The chi-square test and Student’s *t* test were used to identify associations with 30-day device success. McNemar's test was used for the comparison of NYHA functional class and TR changes before and after treatment. A 2-sided *P* value <0.05 was considered statistically significant. All analyses were performed using R version 4.0.4 (R Foundation for Statistical Computing) and SPSS version 29 (IBM).

## Results

A total of 106 patients were included, with a mean age of 76.1 ± 10 years, a median EuroSCORE II (European System for Cardiac Operative Risk Evaluation II) of 4.1 (IQR: 4.7) and a median TriScore of 8.0 (IQR: 17). Additionally, 54.7% (n = 58 of 106) of the patients were female (Table 1). Eighty-nine percent had atrial fibrillation (AF), including 68.9% (n = 73 of 106) with permanent or persistent AF. Prior CIEDs were common (17.9% [n = 19 of 106]). Coexisting significant MR (≥moderate) was observed in 49.1% (n = 52 of 106) of the treated cohort, with a majority having functional MR etiology (83.0% [n = 88 of 106]). Baseline massive or torrential TR was present in 56.6% (n = 60 of 106), and the majority of the TR cases were functional (88.7% [n = 94 of 106]). Over half of the cases had diffuse TR across different tricuspid regions (56.6% [n = 60 of 106]).

**Table 1:** Baseline Clinical Characteristics (N = 106)

Age, y	76.1 ± 10
Female	58/106 (54.7)
Body weight, kg,	58.7 ± 12.9
Body mass index, kg/m^2^	23.1 ± 4.0
Hypertension	57/106 (53.8)
Diabetes	29/106 (27.4)
Atrial fibrillation	94/106 (88.7)
Permanent/persistent atrial fibrillation	73/106 (68.9)
Prior cardiac surgery	28/106 (26.4)
Prior CIED	19/106 (17.9)
Conventional pacemaker	15/106 (14.2)
Leadless pacemaker	3/106 (2.8)
ICD	1/106 (0.9)
EuroSCORE II	4.1 (4.7)
TriScore	8 (17)
NYHA functional class III/IV	52/106 (49.1)
Beta-blocker	76/106 (71.7)
ACE inhibitor/ARB/ARNI	42/106 (39.6)
Aldosterone antagonist	72/106 (67.9)
Lasix	85/106 (80.2)
Thiazide	14/106 (13.2)
Sodium-glucose transporter-2 inhibitors	29/106 (27.4)
MR severity ≥ moderate	52/106 (49.1)
MR Mechanism	
Functional MR	88/106 (83.0)
Degenerative MR	18/106 (16.0)
LVEF, %	56.0 ± 12.3
TR severity	
Moderate	1/106 (0.9)
Severe	45/106 (42.5)
Massive	23/106 (21.7)
Torrential	37/106 (34.9)
TR mechanism	
Functional TR	94/106 (88.7)
CIED-associated TR	6/106 (5.7)
Organic TR	6/106 (5.7)
TR location	
Anteroseptal	35/106 (33.0)
Posteroseptal	11/106 (10.4)
Throughout	60/106 (56.6)
RVSP, mm Hg	47.6 ± 11.8

Values are mean ± SD, n/N (%), or median (IQR).

ACE = angiotensin-converting enzyme; ARB = angiotensin II receptor blocker; ARNI = angiotensin receptor neprilysin inhibitor; CIED = cardiac implantable electronic device; EuroSCORE II = European System for Cardiac Operative Risk Evaluation II; ICD = implantable-cardioverter defibrillator; LVEF = left ventricular ejection fraction; MR = mitral regurgitation; RVSP = right ventricular systolic pressure; TR = tricuspid regurgitation.

Off-label MitraClip was used in 22.6% (n = 24 of 106) of the tricuspid TEER cases in the early experience, which were subsequently replaced by the dedicated TriClip device (Abbott) (77.4% [n = 82 of 106]) when available. A median of 2 clips (IQR: 1 clip) were used, with the majority of clips being XTW clips (57.6% [110 out of all 191 clips] and 73.3% [107 out of all 153 TriClips]) (Table 2). In over half of the cases, the clips were implanted at the AS position only (54.7% [n = 58 of 106]), followed by AS and PS positions (36.8% [n = 39 of 106]). The 3 clipping strategies were adopted evenly (single clip: 35.8% [n = 38 of 106] vs zipping: 27.4% [n = 29 of 106] vs clover: 36.8% [n = 39 of 106]). Notably, combined procedures were performed in 47.2% (n = 50 of 106) of the cases, which included mitral and tricuspid TEER using the MitraClip device (15.1% [n = 16 of 106]), MitraClip and TriClip devices (23.6% [n = 25 of 106]), and other procedures (8.5% [n = 9 of 106]; left atrial appendage occlusion, n = 8; atrial septal or patent foramen ovale occlusion, n = 5; mitral valvuloplasty, n = 1; aortic paravalvular leak closure, n = 1; percutaneous coronary intervention, n = 1; and transcatheter aortic valve replacement, n = 1). Three-dimensional intracardiac echocardiography was used in 9.4% (n = 10 of 106) of cases. The median total procedural time (from vascular access to closure) was 140.5 minutes (IQR: 93.0 minutes) in the whole cohort, and 131.0 minutes (IQR: 92.0 minutes) in the dedicated TriClip cohort. There were no inpatient deaths, myocardial infarctions, or strokes. Single leaflet device attachment was reported in 4.7% (n = 5 of 106). Among patients with CIED in situ, the CIED functions were not affected after tricuspid TEER.

**Table 2:** Procedural Characteristics

	All Tricuspid TEER (n = 106)	TriClip Device Alone (n = 82)
Off-label MitraClip for T-TEER	24/106 (22.6)	0
Number of clips	2.0 (1.0)	2.0 (1.0)
Number of clips used overall	191	153
NTR	20/191 (10.5)	NA
XTR	15/191 (7.9)	NA
G4 NT	0	0
G4 NTW	3/191 (1.6)	3/153 (2.0)
G4 XT	43/191 (22.5)	43/153 (28.1)
G4 XTW	110/191 (57.6)	107/153 (69.9)
Clip location		
AS	58/106 (54.7)	41/82 (50)
PS	9/106 (8.5)	7/82 (8.5)
AS+PS	39/106 (36.8)	34/82 (41.5)
Clip strategy		
Single clip	38/106 (35.8)	26/82 (31.7)
Zipping	29/106 (27.4)	22/82 (26.8)
Clover	39/106 (36.8)	34/82 (41.5)
Combined procedures	50/106 (47.2)	33/82 (40.2)
M/T-TEER using off label MitraClip devices	16/106 (15.1)	0
M/T-TEER using MitraClip/TriClip Device	25/106 (23.6)	25/82 (30.5)
Combined with other transcatheter procedures	9/106 (8.5)	8/82 (9.7)
3D ICE used	10/106 (9.4)	10/82 (12.2)
Procedure time (puncture to closure), min	140.5 (93.0)	131.0 (92)
Inpatient complications		
Death	0	0
Myocardial infarction	0	0
Stroke	0	0
GI complication	2/106 (1.9)	1/82 (1.2)
SLDA	5/106 (4.7)	4/82 (4.9)
Device embolization	0	0
New dialysis	1/106 (0.9)	1/82 (1.2)

Values are n/N (%), median (IQR), or n.

AS = anteroseptal; GI = gastrointestinal; ICE = intracardiac echocardiogram; M-TEER = mitral transcatheter edge-to-edge repair; NA = not applicable; PS = posteroseptal; SLDA = single leaflet device attachment; T-TEER = tricuspid transcatheter edge-to-edge repair; TEER = transcatheter edge-to-edge repair; 3D = 3-dimensional.

**Figure 1:**
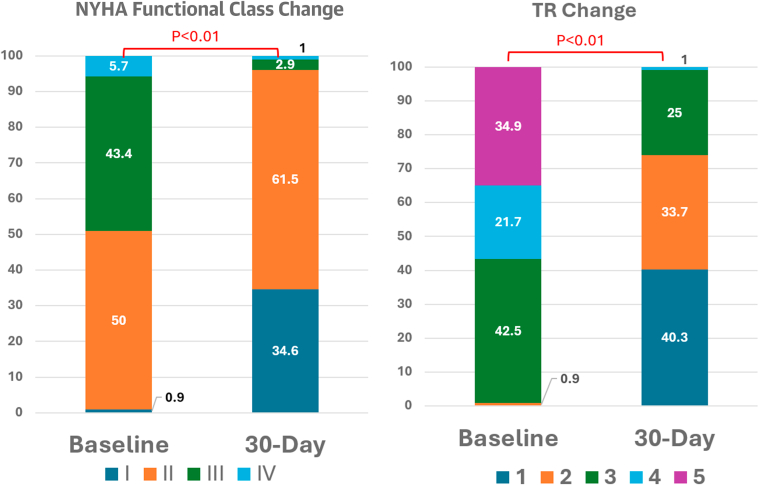
Change in Heart Failure Symptoms and TR From Baseline to 30 Days Significant improvement in heart failure symptoms (NYHA functional class) and the severity of tricuspid regurgitation (TR) were observed after tricuspid transcatheter edge-to-edge repair.

At 30 days, 74.0% (n = 77 of 104; 95% CI: 64.9%-81.5%) achieved device success (≤moderate TR), with 95.2% (n = 99 of 104; 95% CI: 89.2%-97.9%) showing at least 1 grade reduction in TR (Table 3, Figure 1). Heart failure symptoms significantly improved, and NYHA functional class III/IV reduced from 49.1% (n = 52 of 106) to 3.8% (n = 4 of 104) (*P =* 0.001) (Figure 1). The rate of 30-day major adverse events was also rare (1.9% [n = 2 of 106; 95% CI: 0.5%-6.6%] for new-onset renal failure, with no cardiovascular mortality, stroke, or myocardial infarction). There was no statistically significant difference in the rate of 30-day device success between combined procedures vs isolated tricuspid TEER (72.7% [n = 77 of 106; 95% CI: 63.5%-80.2%] vs 75.5% [n = 60 of 80; 95% CI: 64.5%-83.2%]; *P =* 0.825).

**Table 3:** 30-Day Outcomes

	Overall (N = 106)	TriClip Alone (n = 82)
30-d TR severity		
Reduce by 1 grade	99/104 (95.2; 89.2-97.9)	75/80 (93.8; 86.2-97.3)
Reduce by ≥2 grades	72/104 (69.2; 59.8-77.3)	57/80 (71.3; 60.5-80.0)
30-d ≤moderate TR	77/104 (74.0; 64.9-81.5)	60/80 (75.0; 64.5-83.2)
30-d NYHA functional class		
I/II	100/104 (96.2; 90.5-98.5)	81/81 (100; 95.5-100)
III/IV	4/104 (3.8; 1.5-9.5)	0/81 (0; 0-4.5)
30-d MAE	2/106 (1.9; 0.5-6.6)	1/82 (1.2; 0.2-6.6)
Cardiovascular mortality	0	0
Myocardial infarction	0	0
Stroke	0	0
New-onset renal failure	2/106 (1.9; 0.5-6.6)	1/82 (1.2; 0.2-6.6)
Endocarditis requiring surgery	0	0
30-d tricuspid reintervention	0	0

Values are n/N (%; 95% CI).

MAE = major adverse events; TR = tricuspid regurgitation.

On further analysis, patients with baseline nontorrential TR had a higher 30-day device success rate compared with those with baseline torrential TR (82.4% [n = 56 of 68; 95% CI: 88.3%-99.1%] vs 58.3% [n = 21 of 36; 95% CI: 42.2%-72.9%]; *P =* 0.010). Additionally, cases performed in the second half at each center (reflecting increased clinical experience) showed a significantly higher success rate (83.7% [n = 41 of 49; 95% CI: 71.0%-91.5%] vs 65.5% [n = 36 of 55; 95% CI: 52.2%-76.6%]; *P =* 0.044) (Table 4), despite a higher proportion of patients with torrential TR (Supplemental Tables 1 and 2). While TR location, mechanism, combined procedures, and clipping strategy were not associated with device success, successful cases had shorter median procedure times (131.0 minutes [IQR: 84.5 minutes] vs 196.0 minutes [IQR: 141.0 minutes]; *P =* 0.016).

**Table 4:** Comparison of Baseline Characteristics and Procedure Features Between Patients With 30-Day Device Success and Failure

	Device Success (TR ≤Moderate) (n = 77)	Device Failure (TR >Moderate) (n = 27)	*P Value*
Age, y	75.4 ± 11.3	77.9 ± 7.1	0.191
Sex			0.181
Female, %	39	18	
Male, %	38	9	
LVEF, %	57.7 ± 10.8	50.7 ± 15.6	0.043
Presence of CIED			0.384
Yes	12	6	
No	65	20	
RVSP, mm Hg	48.5 ± 12.7	45.3 ± 8.8	0.169
TR mechanism			0.737
Functional	67	25	
CIED associated	5	1	
Organic	5	1	
TR severity			0.010
Nontorrential	56	12	
Torrential	21	15	
TR location			0.988
AS	25	9	
PS	8	3	
Throughout	44	15	
Operator experience			0.044
First half	36	19	
Second half	41	8	
Procedure feature			0.825
Combined Procedure	37	12	
Stand-alone T-TEER	40	15	
Clip strategy			0.903
Single-clip technique	28	10	
Zipping technique	20	8	
Clover	29	9	
Procedure Time, min	148.2 ± 69.9	199.7 ± 92.2	0.021

Values are mean ± SD or n, unless otherwise indicated.

Abbreviations as in Tables 1 and 2.

Compared with published international registries,^10^^,^^12^ the implant success rate (100%) and the rate of 30-day device success (TR ≤moderate) for the dedicated TriClip device were numerically better or comparable (Table 5).

**Table 5:** Comparison of the Initial Asia TriClip Data With Published International Registry Data

	TriClip Asia (n = 82)	bRIGHT Registry (n = 511)	TRILUMINATE (n = 85)
Age, y	76.3 ± 8.6	78.9 ± 7.1	77.8 ± 7.9
Female	56.1	56	66
LVEF, %	57.1 ± 10.2	55.8 ± 10.6	59.4 ± 8.1
Atrial fibrillation	89.0	86.30	86
CIED	18.3	22.50	Excluded
Functional TR	89	90	84
Baseline MR >moderate	41	6	NA
Baseline TR massive/torrential	56.1	88	66
Mean EuroSCORE II, %	6.20	NA	8.60
Implant success	100	NA	100
≤Moderate TR at 30 d	75	77	56
30-d MAE	1.80	2.70	6
Cardiovascular mortality	0	0.80	4
Myocardial infarction	0	0	1
Stroke	0	0.40	0
New-onset renal failure	1.2	1.40	1
SLDA	4.9	3.80	7

bRIGHT = An Observational Real-World Study Evaluating Severe Tricuspid Regurgitation Patients Treated with the Abbott TriClip Device; TRILUMINATE = The Trial to Evaluate Cardiovascular Outcomes in Patients Treated with the Tricuspid Valve Repair System; other abbreviations as in Tables 1 and 3.

Values are mean ± SD or %.

## Discussion

To date, our multicenter registry represents the largest tricuspid TEER experience in the APAC region. Several important findings have emerged from this registry (Central Illustration). First, it showed a high 30-day device success rate (TR ≤moderate) of 74.0% and good safety, with a 30-day major adverse event rate of 1.9% and single leaflet device attachment occurring in 4.7% of cases. Second, the device success rate improves with clinical experience. Third, combined procedures were common in the APAC region.

**Central Illustration:**
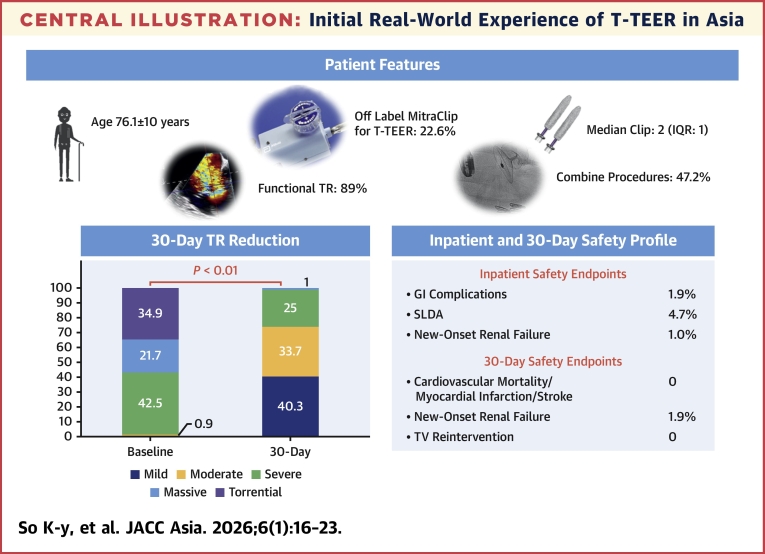
Initial Real-World Experience of T-TEER in Asia The registry included 22.6% of very early experience of tricuspid transcatheter edge-to-edge repair (T-TEER) using off-label MitraClip device. Besides, combined procedures were common. The 30-day tricuspid regurgitation (TR) reduction was significant with low 30-day major adverse events. GI = gastrointestinal; SLDA = single leaflet device attachment; TV = tricuspid valve.

The device success rate (TR ≤moderate) observed in our registry was higher than that reported in the AU^12^ and comparable to the bRight (An Observational Real-World Study Evaluating Severe Tricuspid Regurgitation Patients Treated with the Abbott TriClip Device) registry.^10^ Additionally, heart failure symptoms significantly improved after successful tricuspid TEER, supporting the efficacy of this procedure in the Asian population. Notably, differences were observed in the cohort of patients treated, including less massive or torrential TR and fewer patients classified as NYHA functional class III or IV at baseline. This may be attributed to an increased awareness of the importance of early TR treatment, as the majority of patients included in our study were from the years 2023 to 2024. However, it may also result from more meticulous anatomical and clinical selection for tricuspid TEER in the APAC region, driven by cost concerns, limited physician experience with the device, and hesitancy to perform invasive procedures on very ill patients.

### Importance of experience

The study included 22.6% off-label use of MitraClip, which contributed to heterogeneity in the procedure outcomes. Due to the small sample size in this group, it was challenging to determine whether any differences in outcomes were attributable to the dedicated device design or operator experience. However, our analysis revealed that the device success rate significantly improved with the experience of tricuspid TEER (second half 83.7% vs first half 65.5%; *P =* 0.044). The “learning curve” effect can be explained by improvements in the implanter’s techniques as well as by the enhanced expertise of the imaging team. Additionally, as time progresses, we have developed a better understanding of the anatomy and pathophysiology of TR, which has led to the anatomical exclusion of suboptimal TR reduction. Nonetheless, the availability of other interventional options for the tricuspid valve in the APAC region^6^ also provides alternatives for patients with challenging TEER anatomy who may experience significant residual TR. The recent introduction of 3-dimensional intracardiac echocardiography may also aid in improving procedural efficacy. These advancements contribute to improved outcomes. We believe that systematic training involving implanters, imagers, and multidisciplinary heart team members involved in patient screening and selection is crucial for improving procedural outcomes in the APAC region.

### Combined procedure in Asia

It has been observed that concomitant procedures are frequently performed alongside tricuspid TEER, including mitral TEER, left atrial appendage occlusion, and other transcatheter procedures. Initially, this occurred due to the "drive-by" off-label application of MitraClip in the tricuspid position following a mitral TEER procedure. However, this pattern has persisted after the launch of TriClip. This reflects not only the less stringent regulations regarding combined procedures in the APAC region, but also the preference among APAC patients to address all issues in a single operation rather than staging for another procedure. Additionally, atrial functional MR and TR are prevalent in the APAC region,^6^ which may account for the more frequent combination of mitral and tricuspid TEER with left atrial appendage occlusion.^13^^,^^14^ Further studies will be needed to support the safety and cost-effectiveness of these combined procedures.

### Device selection in the APAC region

Our study found that baseline torrential TR is associated with suboptimal 30-day TR reduction. With the increasing number of devices available in the APAC region to treat TR,^15^^,^^16^ more data are needed to refine the algorithm for anatomical and case selection for these different devices. Head-to-head comparisons and exploration of hybrid therapies to treat TR may be particularly necessary, especially in less favorable anatomical cohorts.

### Study limitations

First, the sample size of the study is small. Although this represented the largest multicenter APAC experience of tricuspid TEER, the small sample size limited the robustness of subgroup analyses and future larger scale study would be needed to confirm the findings of this study. Second, the echocardiographic results were reported by the sites without core lab adjudication. Nonetheless, a standardized classification protocol was used to classify and grade TR, evaluated by experienced interventional echocardiographers at each individual site. However, case-by-case reviews on the mechanism of 30-day device failure were not possible. Third, the short follow-up duration and the lack of a control group in this study limited the ability to accurately assess the long-term effects and generalizability of the findings.

## Conclusions

The early experience with tricuspid TEER in Asia is promising, demonstrating a reasonable device success rate and a high safety profile. Clinical experience is associated with improved device success.

## Funding Support and Author Disclosures

Dr So has served as a physician proctor for Abbott Structural Heart, Boston Scientific, Edwards and Medtronic; and as consultant for Venus Medtech and Jenscare. Drs Meemook and Sung have served as physician proctors for Abbott Structural Heart. Dr Ngernsritrakul has served as a physician proctor (transesophageal echocardiogram proctoring) for Abbott Structural Heart and Boston Scientific. Dr Alex Pui-wai Lee has served as a speaker and consultant for Abbott Structural, Philips, Huihe, and Siemens. All other authors have reported that they have no relationships relevant to the contents of this paper to disclose.
