# Supplementary material for: Initial Real-World Experience of Tricuspid Transcatheter Edge-to-Edge Repair in Asia
Source: JACC Asia. 2025 Sep 24;6(1):16–23. doi: 10.1016/j.jacasi.2025.07.024 (PMC12833604; doi:10.1016/j.jacasi.2025.07.024)
Supplement: Supplementary Material [file mmc1.docx]

**Supplemental Table 1. Comparison of patient and procedure features, and outcomes in patients with torrential versus non-torrential TR.**

|  | **Non-Torrential TR (N=69)** | **Torrential TR**  **(N=37)** | P |
| --- | --- | --- | --- |
| **Age, mean±SD** | 75.6±11.4 | 76.8±7.9 | 0.508 |
| **Gender, Female, %** | 38/69 (55.1%) | 20/37 (54.1%) | 1.000 |
| **LVEF, %, mean±SD** | 57.9±10.7 | 51.6±14.7 | 0.032* |
| **Presence of CIED** | 11/69 (15.9%) | 8/36 (22.2%) | 0.435 |
| **NYHA 3/4** | 30/69 (43.5%) | 22/37 (59.5%) | 0.154 |
| **RVSP, mmHg, mean±SD** | 47.7±12.2 | 47.4±11.3 | 0.909 |
| **TR Mechanism**   - **Functional** - **CIED** - **Organic** | 65  2  2 | 29  4  4 | 0.050 |
| **TR Location**   - **AS** - **PS** - **Throughout** | 24  9  36 | 11  2  24 | 0.329 |
| **Number of Clips, mean±SD** | 1.6±0.6 | 2.1±0.7 | 0.005* |
| **Combined Procedure** | 29/69 (42.0%) | 21/37 (56.8%) | 0.160 |
| **Clip Strategy**   - **Single Clip Technique** - **Zipping Technique** - **Clover** | 29  16  24 | 9  13  15 | 0.673 |
| **Procedure Time, minute, median, IQR** | 123.5 (IQR=69.0) | 195.0 (IQR=70.0) | <0.001* |
| **30-day Device Success** | 56/68 (82.4%) | 21/36 (58.3%) | 0.010* |
| **30-day NYHA I/II** | 68/69 (98.6%) | 34/37 (91.9%) | 0.121 |

*TR=Tricuspid Regurgitation; LVEF=left ventricular ejection fraction; CIED=Cardiac Implantable Electronic Device; AS=Anteroseptal; PS=Posteroseptal; NYHA=New York Heart Association; RVSP=Right ventricular systolic pressure; SLDA=Single Leaflet Device Attachment*

**Supplemental Table 2. Comparison of patient, procedure features, and outcomes between first-half and second-half cases at each site.**

|  | **First Half (N=55)** | **Second Half (N=51)** | **P** |
| --- | --- | --- | --- |
| **Age, mean±SD** | 75.7±11.8 | 76.5±8.6 | 0.675 |
| **Gender, Female** | 29/55 (52.7%) | 29/51 (56.9%) | 0.700 |
| **LVEF, %, mean±SD** | 55.9±14.2 | 55.9±10.3 | 0.972 |
| **Presence of CIED** | 9/54 (16.7%) | 10/51 (19.6%) | 0.802 |
| **NYHA 3-4** | 27/55 (49.1%) | 25/51 (49.0%) | 1.000 |
| **RVSP, mmHg, mean±SD** | 49.7±12.6 | 45.4±10.8 | 0.074 |
| **TR Mechanism**   - **Functional** - **CIED** - **Organic** | 49  3  3 | 45  3  3 | 0.990 |
| **TR Location**   - **AS** - **PS** - **Throughout** | 24  6  25 | 11  5  35 | 0.040* |
| **Torrential TR** | 18/55 (32.7%) | 19/51 (37.3%) | 0.686 |
| **Number of Clips, mean±SD** | 1.7±0.7 | 1.9±0.7 | 0.156 |
| **Combined Procedure** | 26/55 (47.3%) | 24/51 (47.1%) | 1.000 |
| **Clip Strategy**   - **Single Clip Technique** - **Zipping Technique** - **Clover** | 23  15  17 | 15  14  22 | 0.331 |
| **Procedure Time, minute, median, IQR** | 140 (IQR=88.8) | 141 (IQR=128.0) | 0.869 |
| **30-day Device Success** | 36/55 (65.5%) | 41/49 (83.7%) | 0.044* |
| **30-day NYHA I/II** | 51/55 (92.7%) | 51/51 (100%) | 0.119 |

*TR=Tricuspid Regurgitation; LVEF=left ventricular ejection fraction; CIED=Cardiac Implantable Electronic Device; AS=Anteroseptal; PS=Posteroseptal; NYHA=New York Heart Association; RVSP=Right ventricular systolic pressure; SLDA=Single Leaflet Device Attachment*
